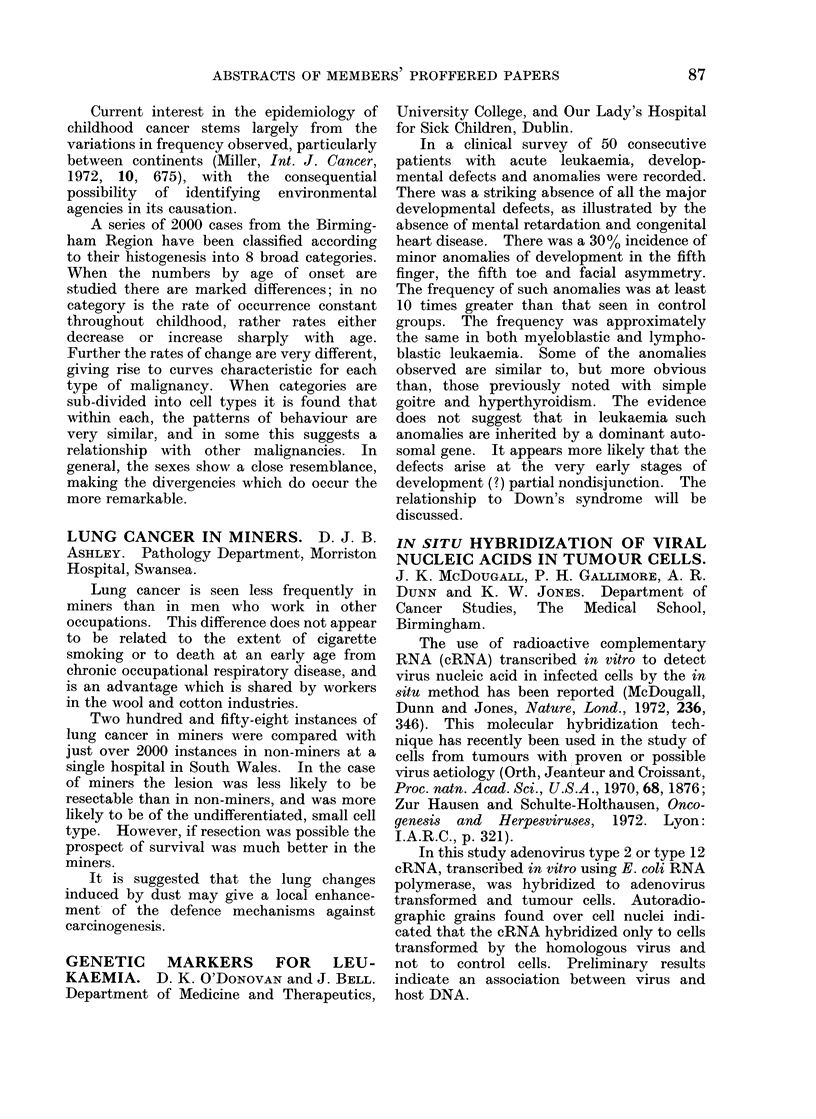# Genetic Markers for Leukaemia

**Published:** 1973-07

**Authors:** D. K. O'Donovan


**GENETIC MARKERS FOR LEUKAEMIA.** D. K. O'Donovan and J. Bell. Department of Medicine and Therapeutics, University College, and Our Lady's Hospital for Sick Children, Dublin.

In a clinical survey of 50 consecutive patients with acute leukaemia, developmental defects and anomalies were recorded. There was a striking absence of all the major developmental defects, as illustrated by the absence of mental retardation and congenital heart disease. There was a 30% incidence of minor anomalies of development in the fifth finger, the fifth toe and facial asymmetry. The frequency of such anomalies was at least 10 times greater than that seen in control groups. The frequency was approximately the same in both myeloblastic and lymphoblastic leukaemia. Some of the anomalies observed are similar to, but more obvious than, those previously noted with simple goitre and hyperthyroidism. The evidence does not suggest that in leukaemia such anomalies are inherited by a dominant autosomal gene. It appears more likely that the defects arise at the very early stages of development (?) partial nondisjunction. The relationship to Down's syndrome will be discussed.